# Paired CRISPR/Cas9 Nickases Mediate Efficient Site-Specific Integration of *F9* into rDNA Locus of Mouse ESCs

**DOI:** 10.3390/ijms19103035

**Published:** 2018-10-05

**Authors:** Yanchi Wang, Junya Zhao, Nannan Duan, Wei Liu, Yuxuan Zhang, Miaojin Zhou, Zhiqing Hu, Mai Feng, Xionghao Liu, Lingqian Wu, Zhuo Li, Desheng Liang

**Affiliations:** Center for Medical Genetics, School of Life Sciences, Central South University, Changsha 410000, China; wangyanchi@sklmg.edu.cn (Y.W.); zhaojunya@sklmg.edu.cn (J.Z.); duannannan@sklmg.edu.cn (N.D.); liuwei@sklmg.edu.cn (W.L.); zhangyuxuan@sklmg.edu.cn (Y.Z.); zhoumiaojin@sklmg.edu.cn (M.Z.); huzhiqing@sklmg.edu.cn (Z.H.); fengmai@sklmg.edu.cn (M.F.); liuxionghao@sklmg.edu.cn (X.L.); wulingqian@sklmg.edu.cn (L.W.)

**Keywords:** CRISPR/Cas9 nickase, gene targeting, Hemophilia B, ribosomal DNA, gene therapy, mESCs, hepatic progenitor like cells, intrasplenic transplantation

## Abstract

Hemophilia B (HB) is an X-linked recessive bleeding disorder, caused by *F9* gene deficiency. Gene therapy combined with the CRISPR/Cas9 technology offers a potential cure for hemophilia B. Now the Cas9 nickase (Cas9n) shows a great advantage in reducing off-target effect compared with wild-type Cas9. In this study, we found that in the multicopy ribosomal DNA (rDNA) locus, the homology directed recombination (HDR) efficiency induced by sgRNA-Cas9n was much higher than sgRNA-Cas9, meanwhile without off-target in six predicted sites. After co-transfection into mESCs with sgRNA-Cas9n and a non-viral rDNA targeting vector pMrnF9, harboring the homology donor template and the human F9 expression cassette, a recombination efficiency of 66.7% was achieved and all targeted clones were confirmed to be site-specific integration of *F9* in the rDNA locus by PCR and southern blotting. Targeted mESCs retained the main pluripotent properties and were then differentiated into hepatic progenitor like cells (HPLCs) and mature hepatocytes, which were characterized by hepatic markers and functional assays. Importantly, the differentiated cells could transcribe exogenous *F9* and secrete coagulation factor IX (FIX) proteins, suggesting active transcription and stable inheritance of transgenes in the rDNA locus. After intrasplenical transplantation in severe combined immune deficiency (SCID) mice, targeted HPLCs could survive and migrate from spleen to liver, resulting in secretion of exogenous FIX into blood. In summary, we demonstrate an efficient and site-specific gene targeting strategy in rDNA locus for stem cell-based gene therapy for hemophilia B.

## 1. Introduction

Hemophilia B is an X-linked congenital bleeding disorder resulting from a deficiency of the functional coagulation factor IX (FIX), encoded by the *F9* gene. Recurrent spontaneous hemarthrosis occurs in severe patients (FIX coagulant activity <1% of the normal value) and moderate patients (FIX coagulant activity 1–5% of the normal value), which is the hallmark of hemophilia B [1]. Hemophilia B affects approximately 1 in 30,000 individuals worldwide [2]. At present, standard care for hemophilia B patients is routine injection of plasma-derived or recombinant FIX concentrates at one to three times per week intravenously [3]. However, the short half-life of FIX, costly repeated infusions, risk of infection, and potential inhibitory antibodies limit its long-term application.

Since the size of the *F9* gene is small and a slight increase of FIX levels will modify the bleeding diathesis, hemophilia B is relatively ideal for gene therapy. Encouragingly, based on viral vectors, especially adeno-associated virus vectors (AAV), several clinical trials have been conducted in small cohorts of hemophilia B patients. Sustained therapeutic expression of FIX coagulant activity was found [4]. However, safety consideration is the main obstacle for wider use of virus vector technologies, like liver toxicity in a few patients and host immune responses to AAV capsid. Up to now, little is known about what drives the immune responses aside from the total vector dose administered and innate immunity to AAV and its clinical implication are largely unknown [5]. Ex vivo therapeutic strategy, on the basis of gene targeting into stem cells via non-viral vectors, would be an attractive choice for gene therapy. This approach allows for the selection and removal of potential hazard mutagenesis and benefits from its less immunotoxicity. Advances in efficiency, specificity, gene expression duration, and safety led to an increased number of non-viral vector products entering clinical trials. One of the key points of this strategy in future clinical applications is to explore a safe genome site for integration of exogenous genes via gene targeting.

The rDNA locus might be an ideal site for exogenous genes. In the vertebrate genomes, there are hundreds of rDNA genes clustered on one or more chromosomes. Theoretically, all copies can serve as candidate targeting sites for transgenes, which may facilitate gene targeting [6]. In addition, human rDNA copy number variations are common among healthy individuals and balanced chromosomal translocations involving rDNA clusters occur without apparent phenotypic change. These properties indicate that the rDNA locus may be an ideal safe locus for transgene integration [7,8]. In our previous studies, exogenous genes have been targeted to the rDNA region and stably expressed in various cell lines by non-viral plasmids, including immortal adult cells and stem cells [9,10,11,12,13].

However, the low targeting efficiency markedly limits the utility of non-viral vectors. Homology-directed gene targeting is extremely rare in mammalian cells, occurring at a frequency of the order of 10^−8^–10^−5^ events per treated cell [14]. In recent years, clustered regularly interspaced short palindromic repeats/CRISPR associated protein 9 (CRISPR/Cas9) has become a powerful tool for genome editing, because it could bring double strand breaks (DSBs) to activate the homology directed recombination (HDR) when there are homologous templates [15]. CRISPR/Cas9 has been successfully applied in many organisms, including mice and humans [16,17,18]. Nevertheless, the off-target effect of CRISPR/Cas9 remains a main concern [19]. To minimize the off-target effect, researchers modified the CRISPR/Cas9 to convert the nuclease into a DNA nickase (CRISPR/Cas9n), which means that the CRISPR/Cas9n can only bring a nick to one of the DNA double strands. Nicked genomic DNA is typically repaired either seamlessly or through high-fidelity HDR, thus the off-target effect of Cas9n is much less than Cas9 [16]. Reports have proven that use of paired CRISPR/Cas9n could result in efficient gene manipulation and less off-target effect [20,21,22].

In this study, we reported for the first time the targeting of human *F9* at the multicopy rDNA locus in mESCs using paired CRISPR/Cas9n and a non-viral targeting vector pMrn. Importantly, we detected exogenous *F9* mRNA and FIX protein in targeted mESCs, and their differentiated hepatic progenitor like cells (HPLCs). Transplantation with *F9* targeted HPLCs into spleens of severe combined immune deficiency (SCID) mice confirmed that HPLCs could migrate into liver and reside to express human FIX.

## 2. Results

### 2.1. Screening for An Efficient rDNA-Targeted sgRNA-CRISPR/Cas9 System

**Figure 1:**
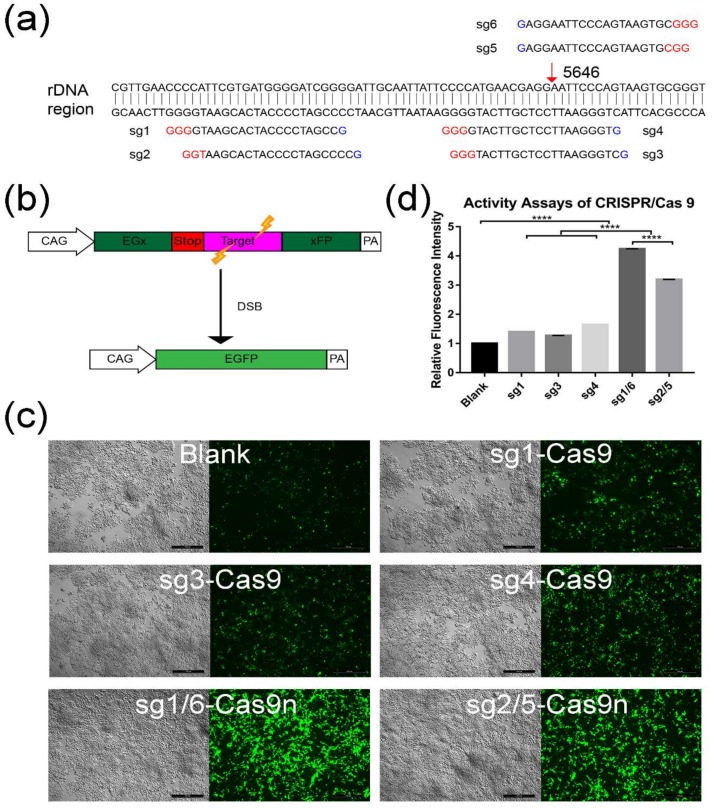
Activity assay for different sgRNA-CRISPR/Cas9 groups. (**a**) Binding position of six different sgRNAs. Red arrow indicates the targeting site in the rDNA region. (**b**) Schematic illustration of activity assays by using the pCAG-EGx-Target-xFP system. The EGFP (enhanced green fluorescence protein) cDNA was divided to two parts, EGx and xFP, in which the “x” represents a repeat fragment of 482 bp. The two parts were separated by the target region (**a**) 77 bp fragment cloned from the rDNA region) along with the stop code. When the sgRNA-CRISPR/Cas9 worked and introduced double strand breaks (DSB), the EGFP cassette was reconstituted by either HDR or SSA. Thus, the green fluorescence could be observed. (**c**) The activity assay for sgRNA-CRISPR/Cas9 was conducted by co-transfecting HEK 293T cells with pCAG-EGx-Target-xFP and sgRNA-CRISPR/Cas9 or sgRNA-CRISPR/Cas9n plasmids (named sg1-Cas9n, sg2-Cas9n, sg5-Cas9n, sg6-Cas9n sg1-Cas9, sg3-Cas9, and sg4-Cas9). sg1/6-Cas9n group means co-transfection with sg1-Cas9n and sg6-Cas9n. In the same way, sg2/5-Cas9n group means co-transfection with sg1-Cas9n and sg6-Cas9n. Blank represented cells only transfected with the plasmid pCAG-EGx-Target-xFP. After 72 h, the green fluorescence of transfected cells was visualized. Scale bar: 500 µm. (**d**) GFP fluorescence intensity of each group was quantified and analyzed with Image J. GraphPad Prism 7.0 was used for calculation and statistics analysis. The relative fluorescence intensity was normalized to blank. (****, *p* < 0.0001).

To facilitate the integration of exogenous genes into the mouse rDNA region via CRISPR/Cas9n, which was a gift from Feng Zhang (Addgene plasmid #42335), we tried to design and screened the most effective sgRNAs around the targeted site—the 5646 locus of musculus ribosomal DNA complete repeating unit (GenBank: BK000964.3). As different sgRNAs may lead to various HDR efficiencies, six sgRNAs (named sg1–sg6) were designed around the same targeting site (Figure 1a, Supplementary Table S1). Four sgRNAs were cloned into the CRISPR/Cas9n nickase (spCas9n) plasmid—namely, sg1-Cas9n, sg2-Cas9n, sg5-Cas9n, and sg6-Cas9n respectively. Among these four sgRNAs, sg1-Cas9n paired with sg6-Cas9n (sg1/6-Cas9n) and sg2-Cas9n paired with sg5-Cas9n (sg2/5-Cas9n) would function together to induce double strand break (DSB) with cohesive ends. Another two sgRNAs (sg3 and sg4) and sg1 were cloned into the Cas9 plasmid as controls, named sg3-Cas9, sg4-Cas9, and sg1-Cas9 respectively.

To evaluate the HDR efficiency mediated by above Cas9n (sg2/5-Cas9n and sg1/6-Cas9n) or Cas9 (sg3-Cas9, sg4-Cas9, and sg1-Cas9) groups, a screening system with co-transfection of plasmid pCAG-EGxxFP was introduced [23]. A 77 bp fragment of target sequence from rDNA, containing the 5646 site, was inserted into the pCAG-EGxxFP. Then the plasmid was co-transfected with sg1/6-Cas9n, sg2/5-Cas9n, sg1-Cas9, sg3-Cas9, and sg4-Cas9 respectively into HEK293T cell. When the target sequence was recognized and DSBs were induced by Cas9 or paired Cas9n, the interrupted EGFP (enhanced green fluorescence protein) cassette can be repaired into a seamless intact gene through either HDR or single strand annealing (SSA) (Figure 1b). Thus, green fluorescence can be observed and visualized by the fluorescence microscopy. Twenty-four hours after co-transfection, green fluorescence was evaluated in each group, including the blank group without Cas9 or Cas9n transfection (Figure 1c). Notably, the fluorescence intensity of all nuclease groups seemed higher than the blank group, especially for the two paired Cas9n groups (Figure 1c). Then the fluorescence intensity was quantified by the software Image J. Statistic results confirmed that both Cas9 and paired Cas9n groups were significantly brighter than the blank control (*p* < 0.0001) (Figure 1d). Compared with the Cas9 groups, the two paired Cas9n groups exhibited markedly higher green fluorescence (*p* < 0.0001), with the highest intensity in sg1/6-Cas9n group (Figure 1d). It suggested that more efficient HDR may occur in sg1/6-Cas9n group, so sg1/6-Cas9n was selected for the following experiments.

### 2.2. Gene Targeting of F9 into the rDNA Region of mESCs by Paired Cas9n and Non-Viral Targeting Vector pMrnF9

**Figure 2:**
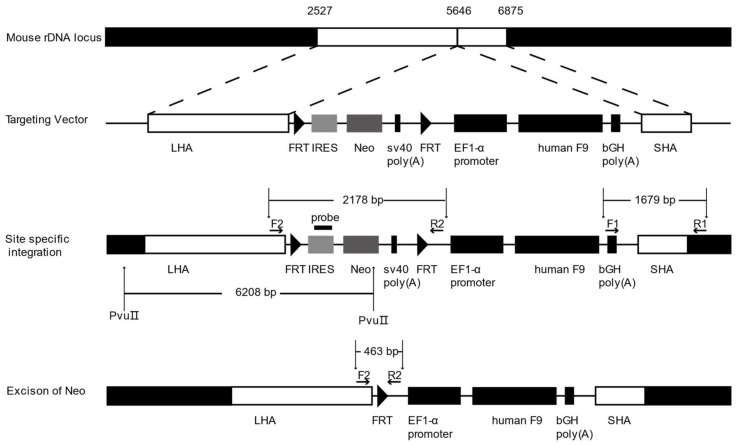
The structure of the non-viral vector pMrnF9 and the schematic diagram of gene targeting into the mouse rDNA locus. LHA, long homologous arm, is homologous to the 2527–5646 region of mouse 45S pre-ribosomal DNA sequence. SHA, short homologous arm, is homologous to the 5646–6875 region. Two short flippase recognition target (FRT) sites flank the *Neo* expression cassette, which links to the internal ribosome entry site (IRES) of encephalomyocarditis virus. By promoter trapping, *Neo* can be transcribed via the in situ rDNA promoter after homologous recombination. A universal promoter EF1-α is utilized to drive the human *F9* expression cassette. Positive targeted cells with site-specific integration of exogenous genes can be filtered by PCR using primer F1 and R1 with a product of 1679 bp. When the genome DNA of targeted clones is digested by PvuIIand detected with the probe homologous to the IRES element, the site-specific clones will show a band of 6208 bp. The *Neo* cassette can be excised by expression of Flpe recombinase. The excised clones can be filtrated by PCR with the primer F2 and R2. A 463 bp band will appear from excised clones while a 2178 bp band remains in original clones.

To target the rDNA locus of mESCs, we designed and constructed the non-viral rDNA-targeting vector, pMrnF9, which would serve as a donor template when co-transfected with sgRNA-Cas9n. The plasmid contains a promoterless neomycin resistance (*Neo*) cassette flanked by flippase recognition target (FRT) sites and an EF1α promoter-driven human *F9* open reading frame (ORF). The two cassettes were flanked by a 3.1kb long homologous arm (LHA) and a 1.2kb short homologous arm (SHA), by which the two expression cassettes could be transferred into the 5464 site of 45S pre-RNA gene via HR. The *Neo* cassette contained an encephalomyocarditis virus internal ribosomal entry site (EMCV-IRES), which enabled resistant gene expression under the control of the endogenous RNA polymerase I (Pol I) promoter upstream after homologous recombination (Figure 2), so that the selected clones could be enriched by this promoter trapping.

**Figure 3:**
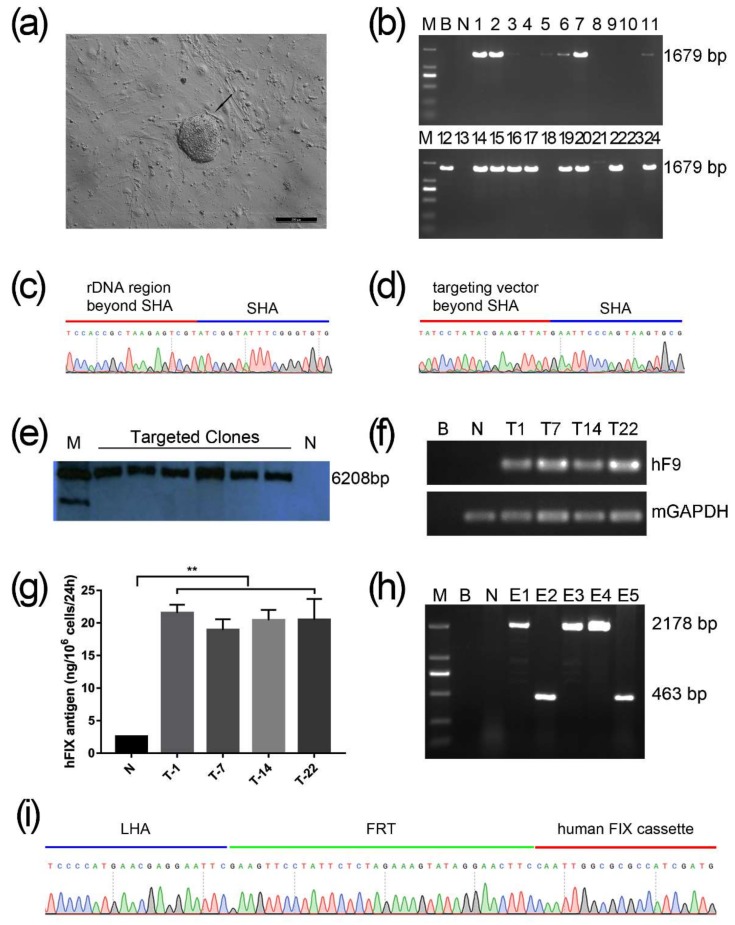
Identification of rDNA site-specific gene targeted mESCs. (**a**) The black arrow indicates a typical mESCs clone after nucleofection with sg1/6-Cas9n and pMrnF9 under selection by G418 for seven days. Scale bar: 200 µm. (**b**) Twenty-four candidate targeted clones were picked and expanded, and followed by genomic DNA extraction. When amplified with primers F1 and R1, the site-specific integrated clones would obtain a 1679 bp band. M, DL2000 DNA Ladder. (**c**,**d**) The PCR products of F1/R1 were sequenced and the results revealed the site-specific integration. (**e**) Southern Blotting for six candidate targeted clones. Positive clones showed an expected band of 6208 bp. M, DNA Molecular Weight Marker II, DIG labeled. (**f**) mRNA of four targeted clones were detected by RT-PCR. (**g**) ELISA for hFIX antigen from the supernatant of targeted mESCs showed the higher level compared with untargeted mESCs (**, *p* < 0.01). (**h**) The *Neo* expression cassette which was flanked by a pair of synclastic FRT elements was removed by secondary nucleofection with the plasmid pCAG-Flpe. When amplified with the primer pair F2 and R2, the excised clones showed a band of 463 bp while 2178 bp indicated non-excision. M, DL2000 DNA ladder. (**i**) The PCR products of F2/R2 were sequenced and the results showed the excision of the *Neo* expression cassette. B, Blank, which represented the template was ddH_2_O; N, Negative control, which was untransfected.

The gene targeting of mESCs was performed by nucleofection with sg1/6-Cas9n and non-viral vector pMrnF9. After selection by G418 at 200 μg/mL for 9–11 days, 24 clones were picked up and expanded (named T1-T24) (Figure 3a). PCR screening with primers F1/R1 showed that 16 out of 24 clones (66.67%) were positive for the expected band of 1679 bp (Figure 3b). The PCR products were then verified by Sanger sequencing, which indicated the junction between the exogenous *F9* cassette and the endogenous rDNA sequence (Figure 3c,d). In addition, six targeted clones were also confirmed by southern blotting using a probe homologous to the IRES element, displaying the gene targeting fragments of 6208 bp (Figure 3e). The top six potential off-target sites of sg1 and sg6 predicted by Cas-OFFinder were detected by PCR followed by Sanger sequencing. As expected, no mutagenesis was found at the six loci (Supplementary Figure S1). Hence, these results indicated the site-specific integration of *Neo* and *F9* expression cassettes into the 5646 site of the mouse rDNA locus without obvious off-target effects.

To determine whether the targeted transgene *F9* could express at the rDNA locus, reverse transcription PCR (RT-PCR) analysis was carried out in the targeted clones T-1, T-7, T-14, and T-22. Results showed that exogenous *F9* can be transcribed in all targeted clones, but not in untargeted mESCs (Figure 3f). Following ELISA for the culture supernatant from the targeted cells found that exogenous human FIX proteins can be synthesized and secreted in all targeted mESCs clones. Their protein levels of human FIX were remarkably higher than the background value from the untargeted control, but no obvious difference among the four targeted clones (Figure 3g). To minimize the effect of exogenous non-therapeutic genes, T-22 clone was used for the excision of the *Neo* cassette by nucleofection again with pCAG-Flpe. Two out of five clones (named E1–E5) were identified to have *Neo* excisions by PCR with primers F2/R2 and then confirmed by Sanger sequencing (Figure 3h,i). Primers and probes used in this study are listed in Supplementary Table S2.

### 2.3. Differentiation of Targeted mESCs into HPLCs and Mature Hepatocytes

**Figure 4:**
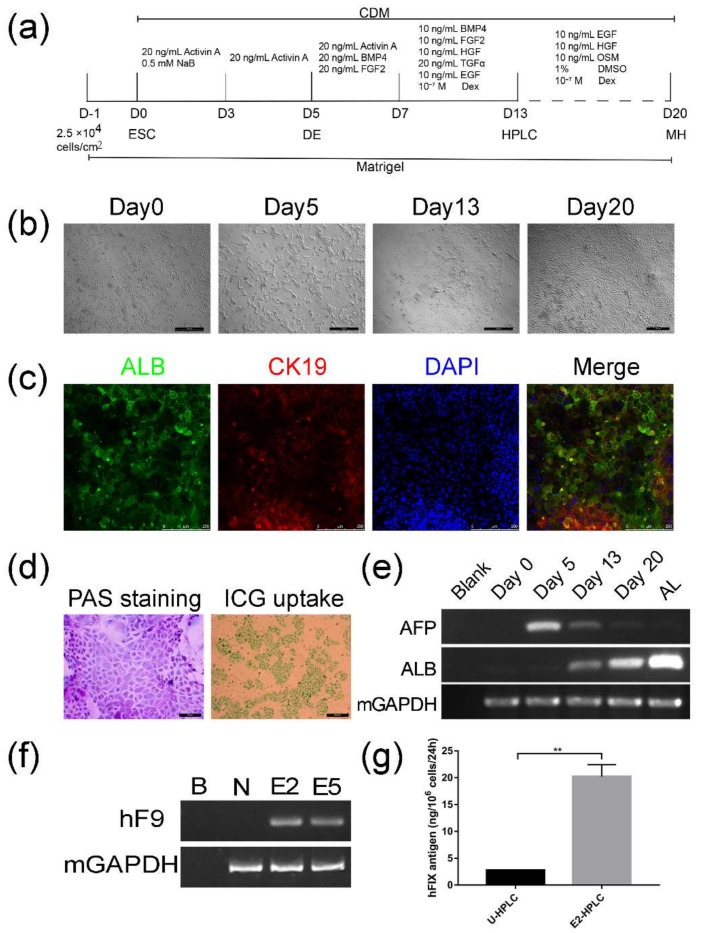
Differentiation of mESCs into HPLCs and detection for exogenous FIX expression. (**a**) Flow chart of the modified protocol for differentiation of mESCs into HPLCs and hepatocytes. (**b**) Dynamic change in cellular morphology during hepatic differentiation (Day0–Day13) and hepatic maturation (Day13–Day20). Scale bar: 500 μm (Day 0, 5, 13) and 200 µm (Day 20). (**c**) Immunofluorescence staining of HPLCs on Day 13 with ALB (Green) and CK19 (Red). Scale bar: 250 μm (**d**) Periodic acid-Schiff’s (PAS) staining and indocyanine green (ICG) uptake assay for the mature hepatocyte like cells on Day 20. The purple-stained and green-stained cells indicate the ability to store glycogen and uptake ICG respectively. Scale bar: 50 μm (PAS staining) and 200 µm (ICG uptake) (**e**) RT-PCR showed the dynamic change of AFP and ALB mRNA during differentiation. (**f**) Differentiated clones on Day 13 were tested by RT-PCR for *F9* transcription. B: Blank; N: untargeted mESCs as negative control. (**g**) Supernatant of untargeted mESCs differentiated HPLCs (U-HPLCs) and E2-HPLCs on Day 13 were gathered to examine the human FIX antigen by ELISA. DE, definitive endoderm. MH, mature hepatocyte. (**, *p* < 0.01).

Since hepatocytes are the main source for FIX synthesis and secretion, we tried differentiate targeted mESCs into HPLCs and hepatocytes. Before the differentiation, the targeted mESCs were verified to be positive for pluripotent markers—including OCT4, SOX2, NANOG, and SSEA1—by immunofluorescence staining (Supplementary Figure S2), indicating that mESCs still kept the pluripotency after rDNA gene targeting and were capable of following differentiation. According to a step-wise protocol (Figure 4a) modified from Li’s protocol [24], cells were first seeded in Matrigel coated 6-well plates at the density of 2.5 × 10^6^ cells/cm^2^ one day before differentiation. Under the continuous induction by replacement with different groups of cellular factors, on Day 13, the differentiated cells grew into monolayer clones, which displayed epithelial morphology and had numerous vacuoles and vesicles in the cytoplasm as well as prominent nucleoli in the nuclei (Figure 4b, Day13). They were confirmed to express hepatic marker albumin (ALB) and cholangetic marker cytokeratin 19 (CK19) by immunofluorescence (Figure 4c), suggesting that the cells had been differentiated into HPLCs, which possess bipotency to mature into hepatocytes or cholangiocytes. 

When HLPCs were further cultured under the condition of hepatic maturation, they were able to differentiate into cells with characteristics of mature hepatocytes, presenting irregular polygon morphology and a large cytoplasm to nuclear ratio (Figure 4b, Day 20). In addition, differentiated cells on Day 20 could metabolize indocyanine green (ICG) and store glycogen (Figure 4d), which are characteristic functions of mature hepatocytes. With the differentiation progress, the dynamic change of transcription levels for alpha-fetoprotein (AFP) and ALB, which indicate early fetal hepatocytes and mature hepatocytes respectively, reciprocally altered as expected (Figure 4e). Importantly, HPLCs derived from targeted clones E2 and E5 were transcriptionally active for human FIX (Figure 4f). The supernatant of HPLSCs derived on Day 13 from untargeted mESCs and the targeted clone E2 were collected for ELISA. Results manifested that human FIX antigen secreted by E2-HPLCs was much higher than for U-HPLCs (Figure 4g, 20.22 ± 2.206 vs. 2.778 ± 0.006603, *n* = 3, *p* < 0.01).

### 2.4. Transplantation of HPLCs in SCID Mice

**Figure 5:**
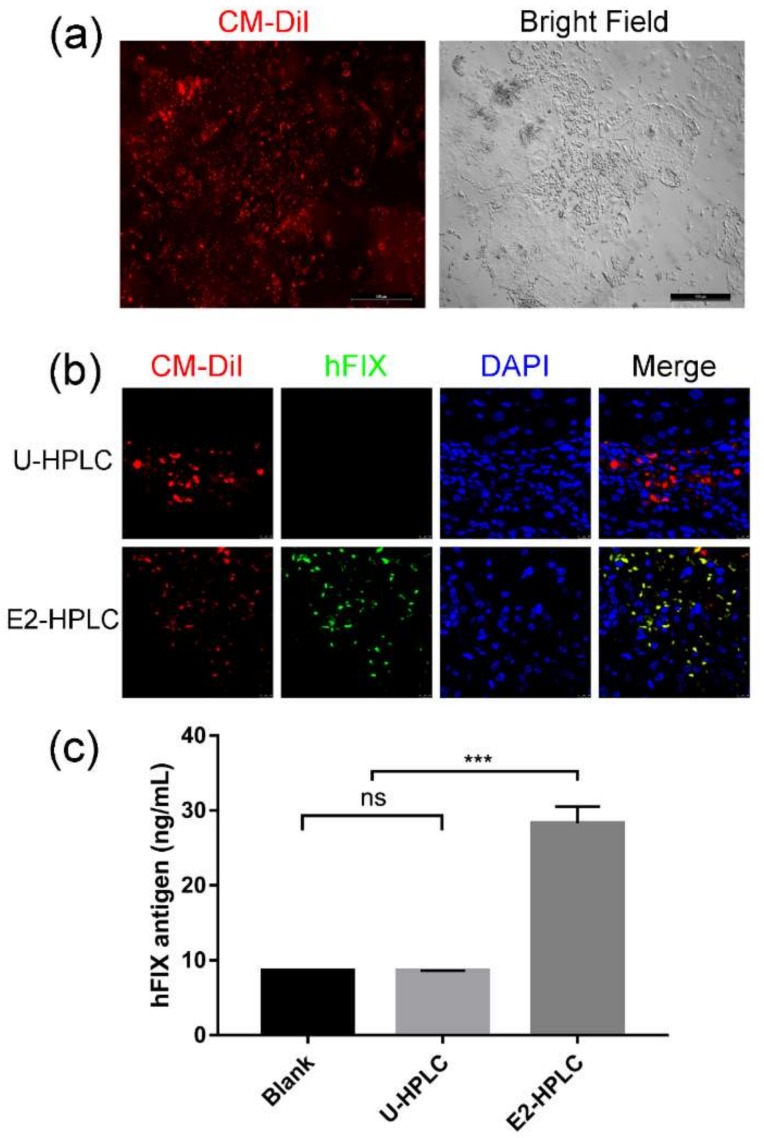
Transplanted HPLCs survived in the liver and produced human Factor IX. (**a**) HPLCs on Day 13 were labeled with CM-DiI. The red fluorescence showed the CM-DiI labeled cells. Scale bar: 500 µm. (**b**) Four weeks after intrasplenic transplantation of HPLCs (Red) into SCID mouse with carbon tetrachloride-induced liver damage. Immunofluorescence staining on liver sections was performed to detect human Factor IX (Green). Scale bar: 10 µm. (**c**) Four weeks after transplantation, the plasma of mice was collected and human Factor IX antigen was detected by ELISA. Blank, mice injected with PBS; U-HPLC, HPLCs differentiated from un-targeted mESCs; E2-HPLC, HPLCs differentiated from *Neo* cassette excised clone E2. ns, not significant; ***, *p* < 0.001.

In order to verify the transplantation feasibility of differentiated HPLCs and whether the cells could express human FIX in vivo, HPLCs were intrasplenically transplanted in SCID mice. Nine SCID mice were randomly divided into three equal groups. To prompt migration of transplanted cells into the liver, liver damage was induced by the administration of carbon tetrachloride for all SCID mice before transplantation. Then the blank control was injected with PBS; the negative control group was transplanted with HPLCs derived from untargeted mESCs (U-HPLCs); the experimental group was injected with HPLCs differentiated from targeted mESCs clone E2 (E2-HPLCs). CellTracker^TM^ CM-DiI, a fluorescent dye which could label live cells, was applied and incubated with differentiated cells before transplantation for tracking cells in vivo (Figure 5a). Four weeks after transplantation, mice were sacrificed to collect blood plasma and livers. The CM-DiI labeled U-HPLCs and E2-HPLCs were both observed in liver sections from transplanted SCID mice, suggesting that the differentiated HPLCs can migrate from the spleen to liver and survive for several weeks. Immunofluorescence of liver sections stained with human FIX antibody revealed that the transplanted E2-HPLCs could also express human FIX (Figure 5b). The collected plasma was analyzed by ELISA. Results revealed that the human FIX antigen of the experimental group was obviously higher than the blank and negative control groups (28.29 ± 2.23 ng/mL vs. 8.52 ± 0.046681 and 8.582 ± 0.02784, *n* = 3, *p* < 0.001) (Figure 5c).

## 3. Discussion

Hemophilia B is a monogenic disorder and even a small increment in plasma FIX activity could alleviate the bleeding symptom. Gene therapy provides hope for a cure for patients with hemophilia B by introducing a functional gene copy of *F9* to repair or compensate the defective gene [1]. Nowadays, gene therapy strategy was usually applied by transduction of target cells with viral vectors. Several clinical trials using viral vectors have been conducted in small cohorts of patients [1,3], but continued concern exists regarding immune response, long-term safety and efficacy. Better solutions are needed to allow for enrollment of AAV seropositive individuals in gene transfer trials for which systemic vector delivery is required [1,5]. The major advantage of using non-viral vectors is bio-safety. However, the application of non-viral based gene therapy has been ignored for a long time in th past due to poor efficiency of delivery, and thereby low transient expression of their transgenes. With the development of technologies in genome editing, stem cells, and gene transfection, an increased number of non-viral vector-based ex vivo gene therapies have been studied and introduced into clinical trials. In this study, we designed and constructed a non-viral targeting vector pMrnF9 and paired CRISPR/Cas9n-sgRNA system to efficiently mediate the exogenous *F9* expression cassette integrating into the rDNA locus of mESCs, exploring the feasibility and advantages of nicking nuclease-facilitated stem cell-based gene therapy for hemophilia B.

It is well known that with the ability to induce DSBs to stimulate the HDR when homologous donor templates were provided, CRISPR/Cas9 has become a widely used tool in gene therapy [25,26]. However, wild-type Cas9 induces numerous undesired mutations that have raised safety concerns for clinical applications. At present, the CRISPR/Cas9 nickase was modified from Cas9 and employed to significantly reduce the off-target. Therefore, CRISPR/Cas9 nickase technology was adopted in this study to further facilitate the gene targeting efficiency in the rDNA region and to limit random insertion. Generally, the specificity and efficiency of the CRISPR/Cas9 or Cas9n technology are dependent on the sgRNA, which complementarily binds to the targeted sequence and links to the Cas9 or Cas9n, ensuring specifically targeted DSB or single nick induction. Therefore, we designed six sgRNAs surrounding the rDNA targeted site for selection by using a pCAG-EGxxFP reporter system. Through comparison of GFP fluorescence intensity, it is easy to determine the HDR efficiency mediated by the sgRNA-Cas9 or sgRNA-Cas9n. After detection, all the paired sgRNA-Cas9n groups were found to mediate higher HDR than the sg-Cas9 groups. This may be explained by either the nick of single strand induced by single sgRNA-Cas9n, or by DSB induced by paired sgRNA-Cas9n contributes to the HDR, since a DNA single strand nick could also induce HDR and the efficiency of paired CRISPR/Cas9n was comparable with CRISPR/Cas9 [20,21,22].

After co-transfection into mESCs with paired sgRNA-Cas9n and pMrnF9, in which pMrnF9 serves as the donor template, 16 out 24 mESCs clones were verified to be rDNA site-specific targeting. The relative targeting efficiency reached about 66.7%, demonstrating that with the assistance of sgRNA-Cas9n in the rDNA region, HDR-mediated gene targeting could be achieved at a high level even in stem cells, compared with our previous studies [27]. Except the main function of sgRNA-Cas9n, the special rDNA properties and the promoter trap strategy should also contribute to promote the relative targeting efficiency. It is reported that the rDNA region exhibits high recombination activity during both meiosis and mitosis [28]. Meanwhile, hundreds of rDNA copies per cell theoretically can all serve as candidate targeting sites for transgenes. Previous research found that the integration frequency of recombinant adeno-associated virus vectors in rat hepatocytes was increased 30-fold by adding a 28S rDNA homologous arm to the vector [7]. By using the promoter trap strategy, the *Neo* is only expressed when it is inserted into a transcriptional active gene and in the proper orientation, so that most of the false-positive resistant clones could be ruled out after drug selection. Moreover, our results also demonstrated that the sgRNA-Cas9n for the rDNA region exhibited a very low off-target with no mutagenesis through sequencing for six potential sites in three different targeted clones. Indeed, we cannot exclude other mutations throughout the whole genome. If necessary, for example in case of subsequent pre-clinical trials, whole genome sequencing should be performed. 

In clinical genetics, variations in the rDNA copy number are common among healthy individuals and balanced chromosomal translocation involving rDNA occurs without any apparent phenotypic effect [29], implying that targeting one or a few copies of rDNA genes might be tolerable to targeted cells. Through the confirmation for the pluripotency of targeted mESCs and its ability to differentiate into HPLCs and mature hepatocytes, our results provided evidence that rDNA site-specific targeting did not change the main properties of mESCs. Therefore, the rDNA locus would be a safe harbor for transgenes. Importantly, all targeted cells, from mESCs to their HPLCs, can transcribe exogenous human *F9* mRNA and secret human FIX proteins, indicating that the transgenes can be expressed and stably inherited in the rDNA region. 

Embryonic stem cells, as pluripotent cells, integrated with an *F9* expression cassette are able to establish a possible inexhaustible source for transplantable HPLCs or hepatocytes. Since transplantation of fetal stem or hepatic progenitor cells into livers of immunodeficient mice resulted in cell expansion and maturation [30,31,32,33], we evaluated the status of targeted HPLCs after transplantation in SCID mice. The engrafted HPLCs with expression of human FIX can be observed in mouse liver four weeks after intrasplenical injection, indicating that the transplanted cells not only remained vital, but also migrated from the spleen to the liver. The human FIX antigen can also be detected in the mouse plasma, with the concentration of 28.29 ± 3.86 ng/mL. Further promotion may be probably accomplished by increasing the injection times and transplanted cell amount. Recent reports of hyperfunctional FIX with R338L or R338A mutations showed the vast potential in future for hemophilia B gene therapy [34,35,36]. Factor IX–R338L is a spontaneous gain-of-function mutation in the factor IX catalytic domain, leading to 8–12 fold increase in specific activity compared to wildtype FIX. Hence, this mutant FIX can be applied for future studies with the similar strategy. In our targeted HPLCs engrafted mice, we did not find tumor formation in the liver or other visible organs at the time of sacrifice. However, additional long-term observations are required to determine the ultimate safety. 

In summary, for the first time, our study demonstrated genome editing in a multicopy region of stem cells using paired sgRNA-Cas9 nickases. Combined with the non-viral vector pMrnF9, as the donor template, we efficiently targeted the exogenous *F9* expression cassette into the rDNA locus of mESCs. The targeted mESCs could be differentiated into HPLCs and hepatocytes, which could stably secrete exogenous FIX protein in vitro. More importantly, the differentiated cells were able to successfully engraft into mice livers, resulting in secretion of exogenous FIX in blood. Our findings suggest that the application of sgRNA-Cas9n in rDNA gene targeting is of great promise in gene and cell therapy for Hemophilia B and other genetic diseases.

## 4. Materials and Methods

### 4.1. Design and Selection for sgRNAs-CRISPR/Cas9 Using Plasmid pCAG-EGx-Target-xFP

sgRNAs for the mouse rDNA locus were designed by Optimized CRISPR Design (available online: http://crispr.mit.edu/). pX330-U6-Chimeric_BB-CBh-hSpCas9 (Cas9) and pX335-U6-Chimeric_BB-CBh-hSpCas9n(D10A) (Cas9n) were gifts from Feng Zhang (Addgene plasmid #42230, #42335). The sgRNA sequences were inserted in the plasmid #42230 or #42335 to generate the constructions sgRNA-CRISPR/Cas9 or CRISPR/Cas9n, respectively.

The plasmid pCAG-EGxxFP was a gift from Masahito Ikawa (Addgene plasmid # 50716), which contained the N-terminal and C-terminal EGFP coding regions overlapping 482 bp under the CAG promoter and a termination codon was placed between the overlapping region (Figure 1a). To complete the construction of plasmid pCAG-EGx-Target-xFP, a 77 bp sequences including the targeting region was inserted between the overlapping region. When the CRISPR/Cas9 works and induces double strand breaks (DSB), the enhanced green fluorescence protein (EGFP) cassette will be reconstituted by either HDR or SSA. The green fluorescence was detected by fluorescence microscopy 72 h after transfection.

### 4.2. Construction of Non-Viral Targeting Vector pMrnF9

The homologous sequences 2527 to 5646 and 5646 to 6875 of mouse 45S pre-RNA genes were amplified by PCR) respectively and then were ligated to the pGEM-T vector, serving as the long homologous arm (LHA) and short homologous arm (SHA). The human FIX expression cassette, IRES element and *Neo* coding sequences were cloned from previously constructed plasmid phrnFIX [9]. The flippase recognition target (FRT) sites were added to PCR primers when the IRES and sv40 (polyA) elements were amplified. Each part was digested and ligated in turn by enzymes to complete the construction of pMrnF9. 

### 4.3. mESCs Culture and Gene Targeting

Mouse embryonic stem cells (R1) were purchased from ATCC and routinely cultured (37 °C 5% CO_2_) on 0.1% gelatin coated dishes in mES medium (MES-M), which contains Knockout DMEM supplemented with 20% FBS, 1 × GlutaMAX™-I, 1 × non-essential amino acid, 1 mM sodium pyruvate, 0.1 mM β-mercaptoethanol and 1000 U/mL human leukemia inhibitory factor (LIF). All reagents were from Life Technologies (Grand Island, NY, USA) except for human LIF (Peprotech, Rocky Hill, NJ, USA).

Transfection of mESCs with the mouse embryonic stem cell Nucleofector^TM^ Kit (LONZA, Walkersville, MD, USA) was performed according to the manufacturer’s manual. Briefly, mESCs were dissociated into single cells with Accutase (Merk Millipore, Watford, UK) and counted. 2 × 10^6^ cells were gathered for nucleofection with 2.5 µg of each CRIPSR/Cas9n plasmid and 5 µg targeting vector pMrnF9 and then plated on 10 cm dishes coated with mouse embryonic fibroblast (MEF) feeding cells. Ten µM of Y27632 was added to the medium to improve the viability of transfected cells. Two days later, G418 was used for selection of resistant clones at the final concentration of 200 µg/mL. About 9 to 11 days after transfection, resistant clones were picked and expanded. Candidate targeted clones were screened by PCR with F1/R1.

### 4.4. Southern Blotting

Genomic DNA was extracted and digested with PvuIIendonuclease (New England Biolabs, Ipswich, MA, USA) at 37 °C overnight and 10 µg of each digested sample was loaded on 0.8% agarose gel followed by electrophoresis at 120 V for 4 h. The samples were then transferred to positively charged nylon membranes, and the DIG labeled Molecular WeightII was used as DNA marker. Thereafter, the membranes were hybridized with DIG Easy Hyb Granules at 42 °C overnight, then washed and detected with CDP-Star Ready to use. The probes were labeled by PCR DIG Probe Synthesis Kit. All reagents and materials except endonuclease PvuIIwere purchased from Roche.

### 4.5. Excision of the Neo Cassette

The plasmid pCAG-Flpe was a gift from Connie Cepko (Addgene plasmid # 13787). *Neo* cassette was excised by transfection of the targeted mESCs with the plasmid pCAG-Flpe. 1 × 10^6^ targeted mESCs were transfected with 5 µg of pCAG-Flpe and then plated on 60 mm-dishes coated with Matrigel^TM^ (BD Biosciences, Erebodegem, Belgium) in MES-M with 10 µM Y27632. Twenty-four hours later, the transfected cells were dissociated and counted, then 1 × 10^3^ cells were replated on MEF feeder cells in 100 mm-dishes. Six to eight days later, clones were picked and expanded.

### 4.6. ELISA for Human FIX Antigen

The medium supernatants were harvested in triplicate from 6-well plates 24 h after medium change. The cells were then washed with PBS, dissociated to single cells and counted. To test the human FIX antigen in mice plasma, the blood was collected and centrifuged. The supernatants were carefully extracted. The ELISA for human Factor IX antigen was performed with paired antibodies for ELISA-Factor IX (Cedarlane Laboratories, Burlington, ON, USA) according to the manufacturer’s instructions. Serial dilution of normal pooled plasma was utilized to construct the reference curves, with the correlation coefficiency (R^2^) greater than 0.980 using a log-log fit.

### 4.7. Differentiation of mESCs into HPLCs and Maturation of HPLCs

The protocol for direct differentiation of mESCs into HPLCs was based on Fuming Li’s protocol with slight modification [37]. The basal differentiation medium called chemically defined medium (CDM), consisted of a 1:1 mixture of neurobasal medium and Dulbecco’s modified Eagle medium (DMEM)/F12 medium supplemented with 0.5 × N2 and 0.5 × B27 supplements, 0.1% bovine serum albumin, 2 mmol/L Glutamax-I, and 0.1 mmol/L 2-mercaptoethanol (all from Invitrogen, Carlsbad, CA, USA). Briefly, mouse ES Cells cultured in CDM added with 1000 U/mL leukemia inhibitory factor (LIF) and 10 ng/mL recombinant human bone morphogenetic protein 4 (BMP4) were digested with Accutase and seeded at the density of 2.5 × 10^4^ cells/cm^2^ on Matrigel^TM^ (BD Biosciences, Erebodegem, Belgium) coated dishes. Twenty-four hours later the medium was changed with CDM with 20 ng/mL Activin A and 0.5 mmol/L sodium butyrate (Sigma-Aldrich, St. Louis, MO, USA). After two days, the cells were cultivated in CDM with 20 ng/mL Activin A for three days. The cells were then replated 5 × 10^4^ cells/cm^2^ on Matrigel-coated dishes in hepatic progenitor specification medium (CDM supplemented with 20 ng/mL Activin A, 20 ng/mL fibroblast growth factor 2 (FGF2)) for two days, following six days in hepatic progenitor expansion medium (CDM supplemented with 20 ng/mL transforming growth factor-α (TGF-α), 10 ng/mL BMP4, 10 ng/mL epidermal growth factor (EGF), 10 ng/mL FGF2, 20 ng/mL hepatocyte growth factor (HGF), and 10^−7^ mol/L dexamethasone (Dex)). During the differentiation, the medium was changed every other day. 

To induce the maturation of HPLCs, the medium was replaced with CDM containing 10 ng/mL HGF, 10 ng/mL EGF, 1% dimethyl sulfoxide (DMSO) and 10 ng/mL Oncostatin M (OSM) for seven days. The medium was renewed every other day. All cytokines used in differentiation and maturation were purchased from PeproTech.

### 4.8. Periodic Acid Schiff Stain and Indocyanin Green Uptake Assay

Periodic acid Schiff stain was carried out with the Periodic Acid Schiff Stain Kit (ScyTek Laboratories, Logan, UT, USA) according to the manufacturer’s instructions for the maturated HPLCs on Day 20. For indocyanin green uptake assay, differentiated cells on Day 20 were first incubated with 1 mg/mL ICG (Sigma-Aldrich, St. Louis, MO, USA). Thirty minutes later, cells were washed with PBS and changed with fresh medium. The PAS stain and uptake of ICG were detected by microscopy. ICG was thoroughly eliminated from the cells 12 h later. 

### 4.9. Intrasplenic Transplantation of HPLCs into SCID Mice

Twenty-four hours before transplantation, six weeks old SCID mice were treated with carbon tetrachloride (CCl_4_, 1 mL/kg) dissolved in mineral oil to induce acute liver damage. 1 × 10^6^ HPLCs derived from untargeted mESCs (U-HPLC) and targeted mESCs with *Neo* cassette excision (E2-HPLC) were labeled with CM-DiI and transplanted into the spleen. For the blank control group, the mice were injected with PBS. Four weeks later, the mice were sacrificed. Liver and plasma were collected for histologic section and ELISA respectively. All animal experiments followed the institutional guidelines approved by the Ethics Committee of Central South University of China (no. 2014031111, 11 March 2014).

### 4.10. Immunofluorescence Staining

Cells were rinsed by PBS three times and then fixed with 4% paraformaldehyde, followed by permeabilization with PBS containing 0.1% Triton X-100. After washing with PBS, cells were incubated with 5% BSA in PBST (PBS + 0.1% Tween 20) for 30 min and then incubated in the diluted primary antibodies in 5% BSA in PBST at 4 °C overnight. On the second day, cells were washed in PBS three times and incubated with the secondary antibody in 5% BSA for 1 h at room temperature in the dark. Cells were plated on cover slips in DAPI (Sigma-Aldrich, St. Louis, MO, USA). Information on antibodies is listed in Supplementary Table S3.

### 4.11. Data Analysis and Statistics

GraphPad Prism 7.0 (GraphPad Software, San Diego, CA, USA) was used for data analysis and statistics. All data are shown as means ± standard error of the means (SEM). Data were compared by unpaired Student’s *t*-test among each treated group and the control or blank groups.

## Supplementary Materials

Supplementary materials can be found at http://www.mdpi.com/1422-0067/19/10/3035/s1.

## Abbreviations

AAV	Adeno-associated virus
CRISPR/Cas9	Clustered regularly-interspaced short palindromic repeats/CRISPR associated protein 9
CDM	Chemically defined medium
DSB	Double strand break
DE	Definitive endoderm
EMCV-IRES	Encephalomyocarditis virus internal ribosomal entry site
FIX	Coagulation factor IX
FRT	Flippase recognition target
HDR	Homology directed recombination
HPLCs	Hepatic progenitor like cells
ICG	Indocyanine green
LHA	Long homologous arm
mESCs	Mouse embryonic stem cells
MH	Maturated hepatocyte
ORF	Open reading frame
PAS staining	Periodic acid-Schiff’s staining
rDNA	Ribosomal DNA
SCID	Severe combined immune deficiency
SHA	Short homologous arm
SSA	Single strand annealing
