# Supplementary material for: Paired CRISPR/Cas9 Nickases Mediate Efficient Site-Specific Integration of F9 into rDNA Locus of Mouse ESCs
Source: Int J Mol Sci. 2018 Oct 5;19(10):3035. doi: 10.3390/ijms19103035 (PMC6213315; doi:10.3390/ijms19103035)
Supplement: Supplementary file 1 [file ijms-19-03035-s001.pdf]

Supplementary Information

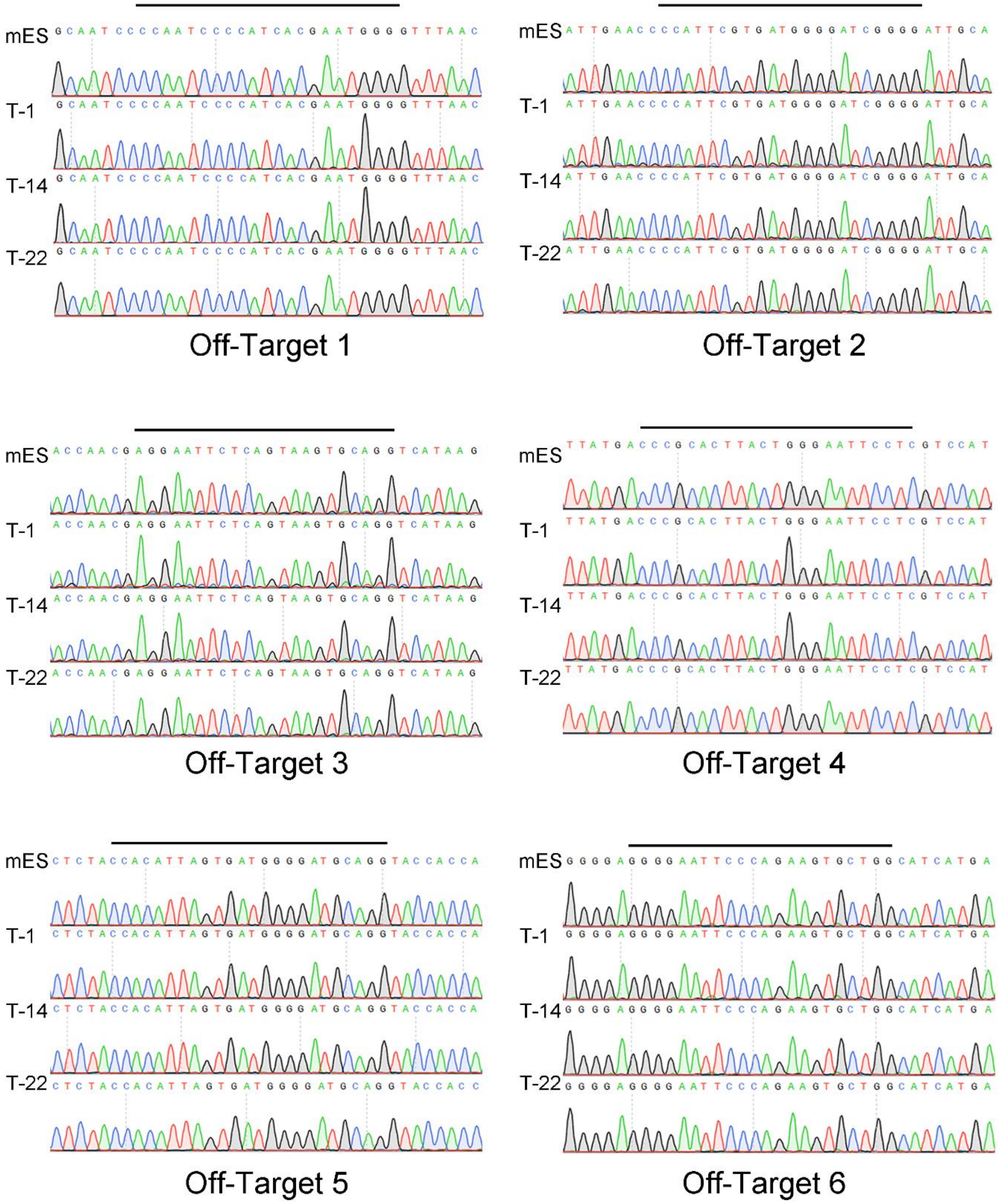

Figure S1. Sanger sequencing for 6 potential off-target sites of sg1 and sg6. Three targeted clones T-1, T-14, T-22 and the untargeted mESCs were analyzed. No indels were detected at the predicted sites. The lines above showed the potential sites.

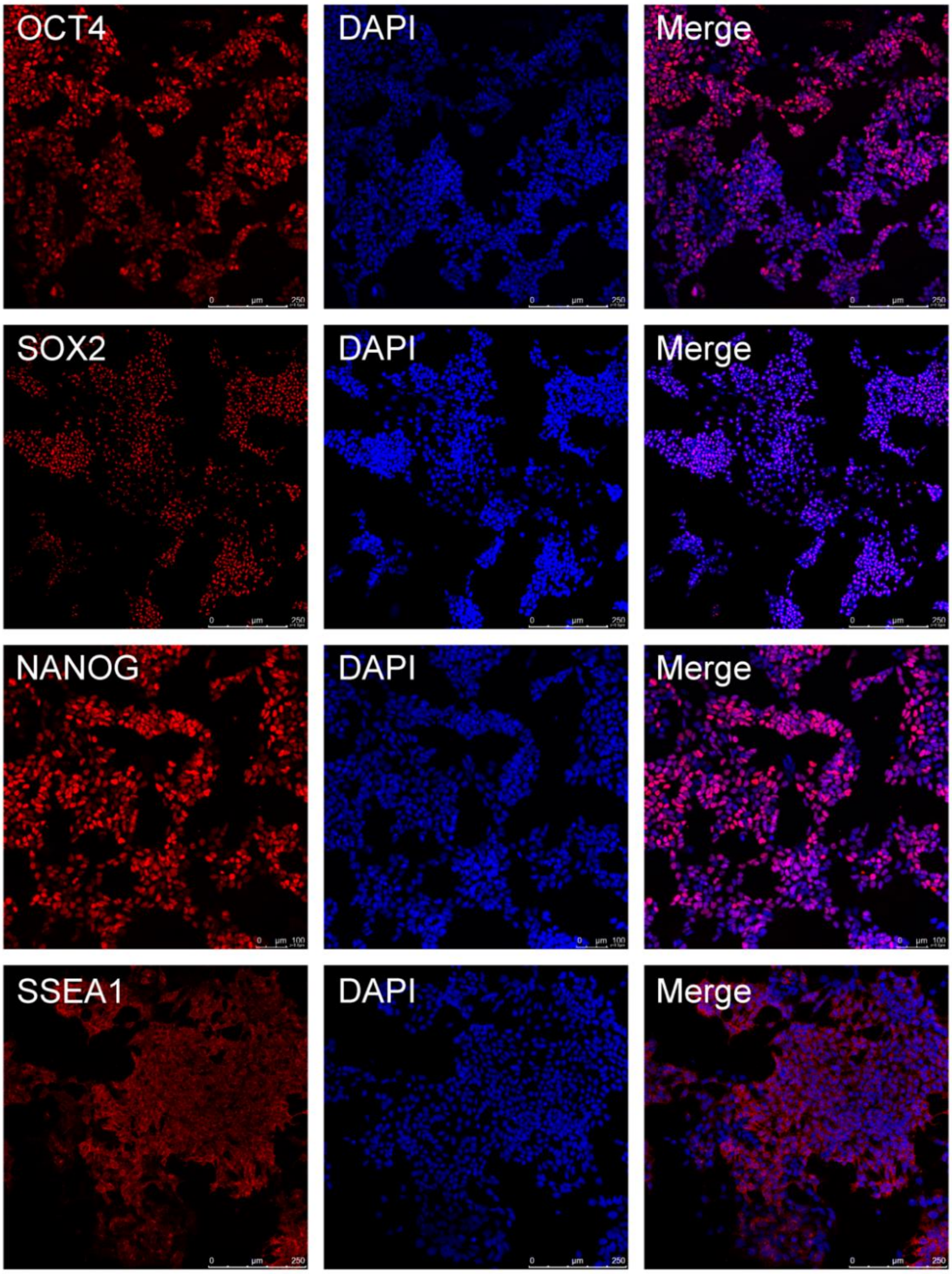

Figure S2. Immunofluorescence staining for targeted mESCs with pluripotent surface markers. Targeted clones expressed the pluripotent surface markers of mouse embryonic stem cells, including OCT4, SOX2, NANOG and SSEA1. Scale bar: 250 μm.

Table S1. SgRNAs designed by Optimized CRISPR Design.

**Name**	**Sequences (PAM)**	
Sg1	GCCGATCCCCATCACGAATG	GGG
Sg2	GCCCCGATCCCCATCACGAA	TGG
Sg3	GCTGGGAATTCCTCGTTCAT	GGG
Sg4	GTGGGAATTCCTCGTTCATG	GGG
Sg5	GAGGAATTCCCAGTAAGTG	CGG
Sg6	GAGGAATTCCCAGTAAGTGC	GGG

Table S2. Primers and probe used in the study

Name	Forward (5′-3′)	Reverse (5′-3′)
F1/R1	GGGCAGGACAGCAAGGGGGAGGAT	CGGATGCGCGGGGGAGACCAG
F2/R2	GGTCGGCGTCCCCCAACTTCTTA	GCAGGGGCGAGTCCTTTTGTATGAAT
Probe	CCCGGAAACCTGGCCCTGTCTT	TGGGGTACCTTCTGGGCATCCTTC
mGAPDH	TGTGTCCGTCGTGGATCTGA	CCTGCTTCACCACCTTCTTGA
hF9	TCTGGAACTGGACGAACCCT	AGACATGTGGCTCGGTCAAC
AFP	GCCACCGAGGAGGAAGTG	AGTCTTCTTGCGTGCCAGC
ALB	GGTGTGTTTCGCCGAGAAGCAC	GGCGGCAGACTCATCGGC

Table S3. Antibodies used in the study

Antibody	Company	catalogue	Application
ALB	R & D	MAB1455	IF (1:200)
CK19	Abcam	Ab52625	IF (1:200)
hFIX	Sigma-Aldrich	F2645	IF (1:100)
OCT4	Abcam	Ab19857	IF (1:250)
SOX2	Abcam	Ab97959	IF (1:500)
NANOG	Abcam	Ab80892	IF (1:100)
SSEA1	Abcam	Ab16285	IF (1:100)
